# Outcomes and Cost-Effectiveness of an EHR-Embedded AI Screener for Identifying Hospitalized Adults at Risk for Opioid Use Disorder

**DOI:** 10.21203/rs.3.rs-5200964/v1

**Published:** 2024-10-14

**Authors:** Majid Afshar, Felice Resnik, Cara Joyce, Madeline Oguss, Dmitriy Dligach, Elizabeth Burnside, Anne Sullivan, Matthew Churpek, Brian Patterson, Elizabeth Salisbury-Afshar, Frank Liao, Randall Brown, Marlon Mundt

**Affiliations:** University of Wisconsin - Madison; University of Wisconsin – Madison; Loyola University Chicago Stritch School of Medicine; University of Wisconsin - Madison; Department of Computer Science, Loyola University; UWisc; University of Wisconsin – Madison; WISC; University of Wisconsin - Madison; University of Wisconsin - Madison; University of Wisconsin - Madison; University of Wisconsin School of Medicine and Public Health, Department of Family Medicine and Community Health; University of Wisconsin - Madison

## Abstract

Hospitalized adults with opioid use disorder (OUD) are at high risk for adverse events and rehospitalizations. This pre-post quasi-experimental study evaluated whether an AI-driven OUD screener embedded in the electronic health record (EHR) was non-inferior to usual care in identifying patients for Addiction Medicine consults, aiming to provide a similarly effective but more scalable alternative to human-led ad hoc consultations. The AI screener analyzed EHR notes in real-time with a convolutional neural network to identify patients at risk and recommend consultation. The primary outcome was the proportion of patients receiving consults, comparing a 16-month pre-intervention period to an 8-month post-intervention period with the AI screener. Consults did not change between periods (1.35% vs 1.51%, p < 0.001 for non-inferiority). The AI screener was associated with a reduction in 30-day readmissions (OR: 0.53, 95% CI: 0.30–0.91, p = 0.02) with an incremental cost of $6,801 per readmission avoided, demonstrating its potential as a scalable, cost-effective solution for OUD care.

**ClinicalTrials.gov ID:**

NCT05745480

## INTRODUCTION

Unintentional overdose involving synthetic opioids and polydrug toxicity has increased exponentially between 2010 and 2022 and remains a critical public health crisis.^1^ Many individuals with nonfatal opioid toxicity and related complications, such as wound infections and endocarditis, frequently engage with hospital care.^2^ From 2018 to 2021, Emergency Department (ED) visits for substance use surged by nearly 40%, with opioids being the second leading cause of these encounters after alcohol.^2,3^ Hospitalized adults with Opioid Use Disorder (OUD) face an elevated risk of overdose or adverse events, making hospitalization an important touchpoint for interventions to prevent rehospitalization or death.^4,5^

Hospital-based addiction care has been shown to increase the adoption of life-saving medications for OUD (MOUD),^6,7^ boost post-hospital treatment engagement,^8,9^ reduce stigma,^10,11^ improve experiences for patients and clinicians,^12^ and lower mortality rates.^13^ However, many hospitals still struggle to provide consistent, high-quality OUD care, resulting in negative patient experiences, delayed presentations, premature discharges, and high morbidity and mortality.^14^ Patients frequently leave the hospital before seeing an addiction specialist, a factor linked to a tenfold increase in overdose rates.^15^ While interprofessional addiction consultation services can enhance MOUD initiation and patient engagement, overall rates remain low.^16^ Identifying and prioritizing these patients for specialized care continues to challenge health systems, leading to inconsistent treatment.^12^

With over 35 million hospitalizations annually, detection rates for OUD are lower than other substances and vary across demographic groups.^17^ The Society of Hospital Medicine recommends that all hospitalized patients with unhealthy opioid use be assessed for OUD.^7^ Although validated tools are available (e.g., Drug Abuse Screening Tool, NIDA Quick Screen, WHO 8-item SUBS, TAPS Tool) ^18–20^, manual screening is resource-intensive, making it difficult to scale for all hospitalized adults. In this digital era, the widespread use of electronic health records (EHRs) and advancements in artificial intelligence (AI) present opportunities for scalable solutions to screen patients at risk for OUD.^21^ EHRs, which routinely capture detailed narratives of substance use, can be leveraged for automated AI-driven screening.^22,23^

We previously developed and validated an AI-driven screener to identify hospitalized patients at risk for OUD, defined as using opioids for non-prescribed or illicit reasons. This screener utilized EHR notes as input data for a convolutional neural network, trained on a reference dataset of scores from the Drug Abuse Screening Tool collected from over 35,000 hospitalizations. The AI screener demonstrated excellent discrimination, calibration, and fairness metrics across multiple sites,^24–26^ paving the way for its integration into a real-time, EHR-embedded workflow at the University of Wisconsin (UW Health).^27^

The primary outcome of this study was to evaluate the proportion of adult hospitalizations that received an Addiction Medicine consultation following the implementation of an AI screener workflow, which identifies patients at high risk for OUD and recommends consultation to the patient’s providers at a hospital with an established inpatient Addiction Medicine service. Using a pre-post study design, we conducted a non-inferiority test to assess whether the addition of the AI screener could match the effectiveness of the pre-period’s provider-driven ad hoc consultation workflow, while providing a more scalable alternative to human-led processes. The secondary outcome included the rehospitalization rate between the periods, along with a cost-effectiveness analysis of the AI screener.

## RESULTS

### AI Screener Implementation and Optimization

The AI screener workflow comprises the AI model, its real-time integration into the EHR, and the recommendation for consultation with the inpatient Addiction Medicine service, delivered as a Best Practice Alert (BPA) to any inpatient provider upon opening the patient’s chart. Prior to the AI screener’s implementation, interviews were conducted with nurse practitioners, residents, and attending physicians from Surgery, Internal Medicine, and Family Medicine to identify potential barriers to using the AI screener. Using the Consolidated Framework for Implementation Research (CFIR)^28^ to guide the interviews and Expert Recommendations for Implementing Change (ERIC)^29^, barriers were addressed through additional educational initiatives, including newsletters and instructional flyers for care teams.

To further optimize utilization, two rapid Plan-Do-Study-Act (PDSA) cycles were conducted between December 2022 and February 2023. The first cycle aimed to reduce the latency of the BPA and minimize the need for ad-hoc addiction consult orders. The AI screener workflow was updated to incorporate notes from the Emergency Department (ED), allowing for an earlier chance of meeting the BPA threshold for a positive screen.

The second PDSA cycle aimed to enhance the information provided to Addiction Medicine providers regarding the reason for consultation. Based on focus group feedback, the BPA was updated to preselect the Clinical Opiate Withdrawal Scale (COWS) for cases where withdrawal symptoms were a potential concern, allowing for the simultaneous ordering of the consult and COWS for the receiving consultant’s review. This update resulted in a significant increase in encounters where both the COWS assessment and Addiction Medicine consult were ordered simultaneously over a four-week period (p < 0.02).

**Figure 1:**
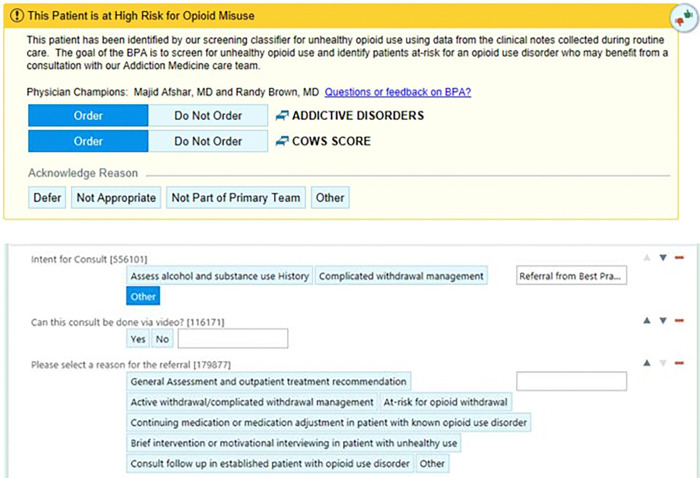
Best Practice Alert for Recommending Addiction Medicine Consult Order and Clinical Opioid Withdrawal Scale (COWS)

Following these cycles, surveys were administered to providers who interacted with the BPA to evaluate its acceptability and utility. All respondents (n = 6) reported that the BPA was a helpful recommendation that did not disrupt their workflow. Based on this feedback, the high-fidelity BPA was implemented hospital-wide. The final version of the BPA is shown in Fig. 1.

### Patient Characteristics During Study Period

**Figure 2:**
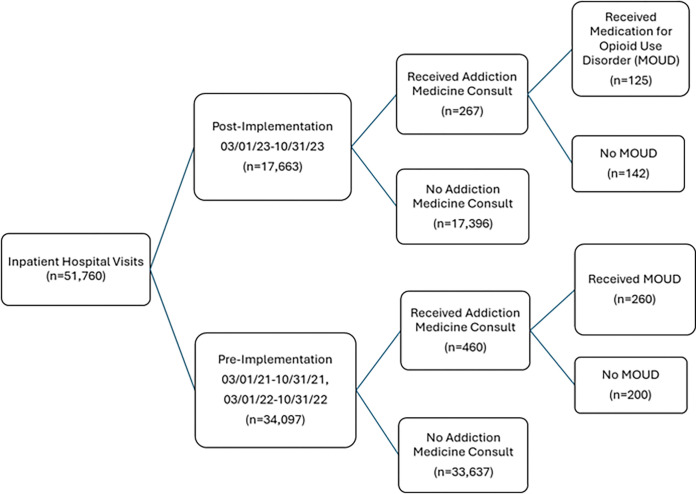
Flow diagram of patients for pre- and post-implementation of Best Practice Alert in the Electronic Health Record for Screening Unhealthy Opioid Use with Associated Interventions

The study included 51,760 adult hospitalizations, with 66% occurring during the pre-intervention period and 34% during the post-intervention period. The AI screener was deployed from March 1, 2023, to October 31, 2023, following two seasonally matched pre-intervention periods (March 1, 2021 - October 31, 2021, and March 1, 2022 - October 31, 2022). A total of 385 Addiction Medicine consults were completed during the entire study period (Fig. 2).

Hospitalized adults in the pre-intervention period were more likely to have chronic conditions such as hypertension, diabetes, and liver disease (p < 0.01) and exhibited higher Elixhauser comorbidity scores and longer hospital stays (p < 0.01). Additionally, a higher proportion of ICD-10 drug misuse diagnosis codes were recorded during the pre-intervention period (p < 0.01). There were no differences in hospital readmission rates between the two periods when examining all hospitalized adults (p = 0.75) (Table 1).

### Post-implementation Addiction Medicine consults are shown regardless of the AI screener

In the subgroup of patients who received an Addiction Medicine consult, demographic characteristics were similar, with few differences in comorbidities, but there was a lower proportion of unhealthy alcohol use and a higher proportion of cannabis use in the post-period (Table 2). In the first 72 hours of admission, the median time to an Addiction Medicine consult order was longer in the post-period than pre-period (11.7 hours vs. 8.4 hours, p < 0.001) but the overall median length of stays was similar (4.6 days vs. 4.2 days, p = 0.79).

### AI Screener’s Performance and BPA Utilization

As each EHR note was entered, the AI screener recalculated the risk score in real-time using all notes available up to that timestamp. A BPA was triggered when a provider opened the patient’s chart if the updated score exceeded the AI model’s predetermined threshold (predicted probability of 0.05). During the post-intervention period, a total of 4,328 BPAs were triggered across 157 hospitalizations identified as high risk by the AI model, with a median of 10 BPAs per hospitalization (IQR 4–23). Among these hospitalized patients, 21.7% (n = 34) received an Addiction Medicine consult directly through the BPA, representing 12.7% (n = 267) of all consults during the post-period. The COWS was initiated in 108 hospitalizations, with 29 cases (26.9%) initiated from the BPA.

The majority of BPAs (90.6%, n = 3,789) were dismissed without a documented reason. The remaining dismissals were attributed to perceived inappropriateness (2.7%, n = 102), deferred action (2.6%, n = 98), canceled consult orders (2.6%, n = 97), involvement of a non-primary team (1.0%, n = 38), and other reasons (0.5%, n = 20).

### Primary Outcome: Addiction Medicine Consults

During the post-intervention period, 1.51% of hospitalized adults received an Addiction Medicine consult compared to 1.35% in the pre-intervention period (z=−1.49, p < 0.001 for non-inferiority). The adjusted odds ratio (aOR) for receiving an opioid-related Addiction Medicine consult post-intervention was 1.09 (95% CI: 0.93–1.28), indicating comparable odds between the two periods after adjusting for age, sex, race/ethnicity, insurance status, and comorbidity score. Similarly, 0.71% of patients received medication for opioid use disorder (MOUD) post-intervention, compared to 0.76% pre-intervention (z = 0.69, p < .001 for non-inferiority), with an aOR of 0.87 (95% CI: 0.69–1.09).

### Secondary Outcome: Rehospitalization Rates

The AI screener’s implementation was associated with a reduction in 30-day readmission rates among patients who received an Addiction Medicine consult, with an aOR of 0.53 (95% CI: 0.30–0.91, p = 0.02). This mixed effects analysis included a random intercept for repeat hospitalizations by the same patient and adjusted for age, sex, race/ethnicity, insurance status, and comorbidity score. Additionally, there was a reduced aOR of 0.67 (95% CI: 0.47–0.94, p = 0.02) for any 30-day post-discharge hospital or ED encounter. Importantly, there was no change in the overall 30-day readmission rate when examining all adult hospitalizations during the same study period (aOR 1.00, 95% CI: 0.94–1.07, p = 0.99). Tables of all adjusted variables and results are shown in the **Supplemental Tables.**

### Cost Effectiveness Analysis

The cost-effectiveness analysis estimated the incremental costs of the AI screener during the 8 months following its implementation (March 1, 2023–October 31, 2023) compared to corresponding 8-month periods in the two years prior. This analysis evaluated the incremental costs in relation to the intervention’s effectiveness in achieving both primary and secondary outcomes.

The development and implementation of the AI screener incurred several costs. Personnel expenses for building the AI model into the EHR included one Principal Analytics Consultant, one Senior Analytics Consultant, one Data Scientist, two Machine Learning Engineers, and one Data Science and Machine Learning Architect. The EHR build began in October 2020 and continued until completion in January 2023. On average, the AI screener development required approximately 0.65 full-time equivalent (FTE) personnel over the 28 months, with total personnel salary and benefits amounting to $234,300. The costs for storage and computing equipment during the development phase were estimated at $109,800. Additionally, training hospital providers and Addiction Medicine consultants on the use of the AI screener incurred a total training cost of $11,600. In total, the development and implementation expenses reached $355,700. The resource costs used for developing and implementing the AI Screener are detailed in **Supplemental Tables**.

The post-implementation incremental costs included ongoing storage and computation expenses, support for Natural Language Processing (NLP) and machine learning components, staff time associated with screening, counseling initiated by the screener, and incremental costs for medications for opioid use disorder (MOUD). The estimated incremental personnel costs for supporting the AI screener during the post-implementation period were $101,400, with additional storage and computing costs of $2,800. Hospital and counseling staff time, as well as medication expenses, contributed another $1,900, bringing the total incremental costs to $106,100 for the post-implementation period. Given that 267 patients received an Addiction Medicine consult during this period, the incremental cost per patient was calculated at $397.

The AI screener’s effectiveness in reducing 30-day readmission was notable, with a 5.8 percentage point difference between pre- and post-implementation rates of 30-day readmission (13.7% pre-intervention vs. 7.9% post-intervention). This equated to an estimated reduction of 15.6 readmissions (95% CI: 2.1–27.1) in the post-intervention period compared to the pre-intervention baseline. Consequently, the incremental cost-effectiveness ratio (ICER) was determined to be $6,801 (95% CI: $3,915–$50,524) for each 30-day readmission avoided, indicating a significant economic benefit of the AI screener in lowering rehospitalization rates.

## DISCUSSION

We evaluated a fully EHR-embedded AI screener in real-time for identifying hospitalized adults at risk for Opioid Use Disorder (OUD) and conducted a comprehensive cost-effectiveness analysis. Our findings demonstrate that the AI screener was non-inferior to the existing practice of ad hoc Addiction Medicine consultations, even after adjusting for significant comorbidities and demographic factors. This suggests that adding the AI-driven workflow can match the effectiveness of an ad-hoc human-led workflow while offering the advantages of automation, particularly in healthcare settings with staff shortages. This study provides valuable insights into the potential for AI to enhance clinical workflows, especially for OUD management

The non-inferiority of receiving an Addiction Medicine consult, with or without MOUD, indicates that the AI screener performed as effectively as the traditional referral workflow initiated by hospital providers. After adjustment for important comorbidities in OUD,^30^ there was no difference in consultations to the Addiction Medicine service or initiation of MOUDs. While the AI screener demonstrated high sensitivity and specificity in identifying patients at risk for OUD,^26^ its translation as an AI-assistive screener embedded in the EHR requires workflow and human factor considerations. The AI screener activated only when a provider opened the patient’s chart, leading to some BPAs being overlooked, not due to the model’s inaccuracy but rather due to provider workflow practices. To address these concerns, we incorporated rapid PDSA cycles with an implementation scientist to improve the AI screener’s utility and prevent alarm fatigue.^31^ Despite the high dismissal rates, most reasons for dismissals were unrelated to the AI model’s predictive accuracy.

The AI screener influenced several aspects of the clinical workflow. In the post-intervention period, Addiction Medicine consults were ordered later during hospitalization, despite no change in the overall length of stay. Unlike manual screening or ad hoc consultations, the AI screener consistently triggered the BPA until provider action was taken, with a median of 10 BPA activations per patient hospitalization, accounting for approximately 13% of all Addiction Medicine consults. This persistent alerting kept providers informed about patients’ OUD needs, serving as a reminder of the Addiction Medicine service that offers pharmacologic and nonpharmacologic interventions (e.g., counseling, harm reduction services) not typically provided by primary care teams before discharge. This approach may have contributed to the associated reduction in 30-day readmission rates.

Recent evidence supports that initiating MOUD during hospitalization and utilizing Addiction Medicine consultation teams improve post-hospital health outcomes.^6,13,32,33^ In our study, the addition of an AI screener was associated with a significant reduction in 30-day readmission rates among patients with OUD in the post-intervention period. This associated reduction remained consistent whether using the CMS definition of readmission or considering all post-discharge encounters, including ED and observation visits. Importantly, no major systemic changes occurred during the study period that would explain this improvement, suggesting that the AI screener was a factor in reducing readmissions, even after controlling for confounders.

The cost-effectiveness analysis revealed that the incremental cost per readmission avoided was approximately $6,800, a substantial saving considering the average cost of a 30-day readmission is estimated at $16,300. ^34^ Patients with OUD typically have longer hospital stays, higher comorbidity burdens, and more disadvantaged payor mixes, making the cost of care for these patients significantly higher than average. The AI screener offers an opportunity to identify and intervene with these high-risk individuals who are disproportionately affected by the overdose crisis, ^35–37^ potentially preventing readmissions. This study adds to the growing evidence of the cost-effectiveness of Addiction Medicine consult services. ^38,39^ With total start-up costs of approximately $350,000 for implementing the BPA in a real-time AI workflow, such AI tools could prove economically viable over time. This study is among the first to evaluate the cost-effectiveness of AI-assisted technology for hospital providers.

Several limitations should be acknowledged in this study. First, it was conducted within a single health system using a specific EHR platform, which may limit generalizability to other systems with different addiction services or patient populations. Additionally, the quasi-experimental design, which compared pre- and post-intervention periods, is susceptible to temporal confounders. Despite conducting a priori sample size calculations and matching study periods for seasonality, residual confounding may still have influenced the results. Lastly, the cost-effectiveness analysis was conducted from a healthcare system perspective and did not account for broader societal costs or benefits. While the ICER per readmission avoided was favorable, the financial investment required might challenge health systems with limited resources.

In conclusion, the addition of an AI-screener to recommend and Addiction Medicine was non-inferior to prior ad hoc consultations and was associated with reduced readmission rates with potential cost savings. Given the increasing adoption of AI-driven clinical decision support tools in EHRs and improved software interoperability, the economic impact of such AI-driven interventions warrants further investigation.^40^ This study demonstrates the potential of an EHR-embedded AI workflow for a hospital-wide screening program and offers valuable insights for other health systems seeking to use AI to optimize services, particularly for vulnerable populations such as patients with OUD.

## METHODS

### Hospital Setting and Study Period

The AI screener was implemented in the EHR (Epic Systems Corp, 2023) at the University of Wisconsin (UW Health) University Hospital across all adult inpatient wards. The study evaluated the screener’s effectiveness in hospitalized adults (aged ≥ 18 years) using a pre-post quasi-experimental design. This design compared seasonally matched pre-intervention periods (March 1, 2021 - October 31, 2021, and March 1, 2022 - October 31, 2022) with a post-intervention period (March 1, 2023 - October 31, 2023). The patient flow chart is shown in Fig. 2. The study was deemed exempt by the University of Wisconsin Institutional Review Board and registered on ClinicalTrials.gov (NCT05745480). The full study protocol was published previously.^27^ This study adhered to the CONSORT-AI guidelines^41^ to ensure comprehensive and transparent reporting of the AI-driven intervention, as outlined in the **Supplemental materials.**

### Pre-Intervention Period: Usual Care with Ad Hoc Addiction Consultations

The Addiction Medicine inpatient consult service at UW Hospital was established in 1991. Among substances, only alcohol screening had a formal process, using the Alcohol Use Disorders Identification Test–Concise (AUDIT-C).^42^ During the pre-intervention period, consultations for patients with opioid use disorder (OUD) were initiated at the discretion of the primary provider, with orders placed in the EHR. The Addiction Medicine consult team, consisting of three Addiction counselors and four physicians, conducted the consultations. Each completed consultation could include: (1) opioid use assessment and brief behavioral intervention; (2) initiation, continuation, or adjustment of medication for opioid use disorder (MOUD); (3) harm reduction services; and/or (4) referral to community-based SUD treatment.

### Post-Intervention Period: AI Screener as a Clinical Decision Support Tool embedded in the EHR

To integrate the AI model into a screening tool as a Best Practice Alert (BPA), the Applied Data Science team at UW Health developed a comprehensive Development and Operations (DevOps) framework. This system, described in detail previously,^27^ uses Health Level 7 (HL7) standards to exchange patient notes from the EHR. Data are stored in a HIPAA-secure Microsoft Azure cloud data lake and processed using a Natural Language Processing (NLP) engine to extract key features as coded medical concepts from EHR notes, utilizing the Unified Medical Language System from the National Library of Medicine. These medical concepts are then input into a trained convolutional neural network (CNN) model, which has a vocabulary of 37,317 medical concepts and 12.5 million parameters. The trained model is available at https://git.doit.wisc.edu/smph-public/dom/uw-icu-data-science-lab-public/smart-ai.

**Figure 3:**
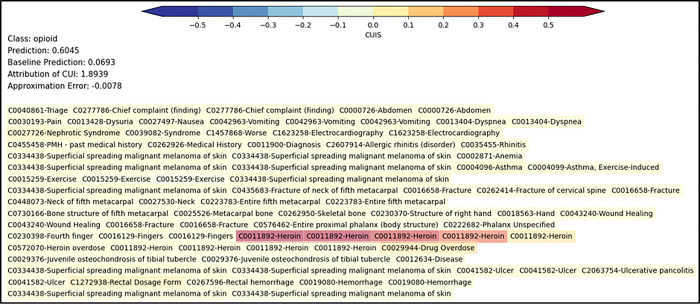
Single-note illustration of AI screener

The AI model’s predictions were assessed using Integrated Gradients (IG) to understand feature contributions from the medical concepts.^43^ IGs operate by comparing the model’s prediction on a given input with a baseline input that represents the absence of features (neutral state). This method calculates the gradients of the model’s output relative to the input, tracing a path from the baseline to the actual input. By integrating these gradients, attribution scores were derived to highlight the contribution of each text feature to the final prediction. For this analysis, padded tokens were used as the baseline input for medical concepts. The analytics team examined the IGs post-hoc to monitor the model during deployment. An example note displaying medical concepts and their attribution scores is shown in Fig. 3.

### Deidentified Emergency Department Triage EHR Note from the encounter:

[xx] yo male presents to the ED with chief complaint of LUQ abd pain since this AM. Also reports dysuria. +nausea. Denies emesis. Associated SOB. Dx with nephrotic syndrome recently reports sx are getting worse. Appears uncomfortable. EKG ordered. To wait in WR for room placement at this time. Past Medical History: Diagnosis Date Allergic rhinitis; Anemia; Asthma, exercise induced; Closed displaced fracture of neck of fifth metacarpal bone of right hand with routine healing [xx/xx/xxxx]. Closed displaced fracture of proximal phalanx of left ring finger [xx/xx/xxxx]; Heroin overdose [xx/xxxx]; Osgood-Schlatter’s disease; Ulcerative pancolitis with rectal bleeding [xx/xxxx]

Processed in Natural Language Processing Engine to medical concepts and fed into AI model with resultant output:

A visualization example demonstrates the individual-level clinical utility of the extracted medical concepts as concept unique identifiers (CUIs) mapped from the note to the Unified Medical Language System metathesaurus at the National Library of Medicine. Integrated Gradients assigns an importance score to each input CUI by approximating the integral of the gradients of the AI model’s output to the inputs. This method compares the model’s prediction on the actual input with a baseline generic note with random CUIs, which typically represent the neutral state. The gradients are calculated at several points along the path from the baseline to the actual input. The integral of these gradients gives an attribution score for each CUI, indicating its contribution to the final prediction. Warmer background colors indicate an increased likelihood of unhealthy opioid use, while cooler colors reflect a decreased probability. The final predicted probability was 0.6753, which is above the threshold for a screen positive; whereas a baseline note with generic tokens for medical concepts had an expectedly lower probability of 0.0693. The process of approximating the integral introduces some approximation error, which depends on the number of steps used in the integration process. More steps typically reduce this error but increase computational complexity. In our case, the CUIs with higher attribution scores significantly influenced the predicted probability of unhealthy opioid use, highlighting their clinical relevance.

The AI-generated scores were transmitted back to the EHR, triggering the BPA when a provider accessed a patient’s chart if the AI score exceeded a predetermined threshold. The alert recommended a consult and preselected the Clinical Opiate Withdrawal Scale (COWS) order set (Fig. 1). For clarity, the complete CDS tool, including the AI model, DevOps integration, and BPA within the EHR, is referred to as the ‘AI Screener’.

### Statistical Analysis

The analytic cohort included all adult hospitalizations during the study period. The primary outcome was the proportion of patients receiving an inpatient Addiction Medicine consult, evidenced by a completed consult note in the EHR. Hypothesis testing used independent sample z-tests to compare intervention effects, with non-inferiority assessed at a one-sided significance level of 0.025. We hypothesized that the AI screener would be as effective as usual ad hoc consultations. Based on prior analyses, we anticipated a 3% prevalence of OUD among inpatients. To achieve 85% power to detect a 0.75% increase in the post-intervention period, we required a sample size of 12,500 patients (10,000 from the pre-intervention period and 2,500 from the post-intervention period). The non-inferiority margin was set at 0.5%, corresponding to a post-intervention proportion of less than 2.5% under the null hypothesis of inferiority.

Secondary outcomes included the 30-day unplanned hospital readmission rate among those receiving an Addiction Medicine consult. The Centers for Medicare and Medicaid Services’ (CMS) criteria for unplanned hospital readmissions were applied.^44^ Sensitivity analyses also included all post-discharge hospital encounters, such as observation stays and ED visits, since nearly one in five rehospitalizations could be missed if non-inpatient encounters were excluded.^45^

A mixed-effects logistic regression model was used to estimate the odds ratio for receiving a consult in the post-intervention period, adjusting for potential confounders, including age, sex, race/ethnicity, insurance status, and comorbidity score. Ther random intercept accounted for repeated hospitalizations in the same patient. The Elixhauser comorbidity score was used to measure overall patient health status with comorbidities.^46^ This score accounts for 31 different comorbidities to predict in-hospital mortality, length of stay, and other outcomes.

### Cost Analysis

The economic evaluation considered: (1) opportunity start-up costs for implementing the AI screener; (2) incremental medical costs comparing usual care to the addition of the AI screener; and (3) ongoing costs for administering and maintaining the AI screener. All costs were adjusted to 2024 US dollars and analyzed from a healthcare system perspective.

The start-up costs for establishing the AI screener included expenses related to supporting the NLP and machine learning components, building the BPA within the EHR, and training healthcare professionals on how to use the tool. To identify the administration and maintenance costs associated with the AI screening workflow changes, we employed the following approach: (1) conducting in-depth interviews with hospital administrators; (2) performing activity-based observations of healthcare personnel using the AI screener; and (3) querying the clinician messaging system within the EHR. We used average hospital compensation rates to value healthcare personnel time costs. Incremental costs between usual care and the AI screener were determined by calculating medical care costs before and after the implementation of the AI screener. These costs included those associated with the hospitalization stay and all subsequent medical expenses for the 30 days following hospital admission using the hospital billing records for all the hospitalized adults during the study period.

The cost-effectiveness analysis was reported in terms of the incremental cost-effectiveness ratio (ICER) per additional patient who received substance use treatment. For this study, the ICER was calculated as the difference in intervention costs between the pre- and post-implementation periods, divided by the difference in intervention effectiveness between these periods, as measured by 30-day hospital readmission and any 30-day post-discharge hospital or ED encounter.

## Figures and Tables

**Table 1:** Patient Characteristics and Demographics for all hospitalized patients

	All Hospitalized Patients (n = 35494)	Pre-Period (n = 23586)	Post-Period (n = 11908)	P-value

Age, median (IQR)	62 (47–73)	62 (47–73)	63 (47–73)	0.122

Male sex, n (%)	18360 (51.7)	12229 (51.8)	6131 (51.5)	0.519

Race + Ethnicity,n(%)	30718 (86.5)	20402 (86.5)	10316 (86.6)	0.361
NH White	2058 (5.8)	1365 (5.8)	693 (5.8)	
NH Black	1086 (3.1)	724 (3.1)	362 (3.0)	
Hispanic	270 (0.8)	167 (0.7)	103 (0.9)	
Mixed	1362 (3.8)	928 (3.9)	434 (3.6)	
Other/Unknown				

Insurance, n (%)	15199 (42.8)	9691 (41.1)	5508 (46.2)	<.001
Medicare	3889 (11.0)	2561 (10.9)	1328 (11.1)	
Medicaid	13716 (38.6)	9335 (39.6)	4381 (36.8)	
Private	2690 (7.6)	1999 (8.5)	691 (5.8)	
Other				

Comorbidities, n(%)				

Hypertension	2983 (8.7)	2090 (9.2)	893 (7.8)	< .001

Renal Failure	1601 (4.7)	1135 (5.0)	466 (4.0)	< .001

Neurologic	1522 (4.4)	1079 (4.8)	443 (3.8)	< .001

CHF	2692 (7.9)	1838 (8.1)	854 (7.4)	0.027

Diabetes	3493 (10.2)	2474 (10.9)	1019 (8.9)	< .001

Liver disease	974 (2.8)	697 (3.1)	277 (2.4)	< .001

Chronic lung disease	747 (2.2)	512 (2.3)	235 (2.0)	0.199

Psychiatric disorders	507 (1.5)	354 (1.6)	153 (1.3)	0.095

Depression	1018 (3.0)	710 (3.1)	308 (2.7)	0.020

Alcohol Misuse	835 (2.4)	582 (2.6)	253 (2.2)	0.038

Drug Misuse	236 (0.7)	179 (0.8)	57 (0.5)	0.002

AIDS	46 (0.1)	32 (0.1)	14 (0.1)	0.644

Elixhauser score, mean (SD)	2.87 (4.97)	2.98 (5.10)	2.63 (4.68)	< .001

Length of Stay, mean (SD)	5.25 (7.62)	5.34 (8.18)	5.05 (6.35)	< .001

Readmission to hospital, n (%)	4931 (13.9)	3267 (13.9)	1664 (14.0)	0.753

Disposition, n (%)	29180 (82.2)	19258 (81.7)	9922 (83.3)	< .001
Home	1084 (3.1)	770 (3.3)	314 (2.6)	
Death	4949 (13.9)	3383 (14.3)	1566 (13.2)	
LT RC / ST PA	235 (0.7)	142 (0.6)	93 (0.8)	
AMA	46 (0.1)	33 (0.1)	13 (0.1)	
Other				

Comparisons across all variables were significant with a p-value < 0.01; AMA = against medical advice; AIDS = acquired immunodeficiency syndrome; LT RC/ST PA = Long term residential care or short term post acute care; CHF = congestive heart failure; NH = non-Hispanic; Mixed = Asian, Native American or Alaskan Native, Native Hawaiian or Other Pacific Islander, Other, or Refuse/Unknown

**Table 2:** Characteristics and Outcomes for Hospitalizations receiving an Addiction Medicine consult in patients at risk for an Opioid Use Disorder

	All Patients (n = 727)	Pre-Period (n = 460)	Post-Period (n = 267)	P-value

Age, median (IQR)	41 (32–55)	40 (32–54)	41 (32–56)	0.284

Male sex, n (%)	465 (64.0)	284 (61.7)	181 (67.8)	0.101

Race + Ethnicity,n(%)	589 (81.0)	385 (83.7)	204 (76.4)	0.295
NH White	97 (13.3)	53 (11.5)	44 (16.5)	
NH Black	16 (2.2)	9 (2.0)	7 (2.6)	
Hispanic	2 (0.3)	1 (0.2)	1 (0.4)	
Mixed	23 (3.2)	12 (2.6)	11 (4.1)	
Other/Unknown				

Insurance, n (%)	123 (16.9)	75 (16.3)	48 (18.0)	0.447
Medicare	388 (53.4)	241 (52.4)	147 (55.1)	
Medicaid	171 (23.5)	117 (25.4)	54 (20.2)	
Private	45 (6.2)	27 (5.9)	18 (6.7)	
Other				

Comorbidities, n(%)				

Hypertension	40 (5.5)	33 (7.2)	7 (2.6)	0.009

Renal Failure	6 (0.8)	4 (0.9)	2 (0.8)	0.863

Neurologic	46 (6.3)	36 (7.8)	10 (3.8)	0.029

CHF	47 (6.5)	25 (5.4)	22 (8.2)	0.138

Diabetes	31 (4.3)	18 (3.9)	13 (4.9)	0.539

Liver disease	63 (8.7)	49 (10.7)	14 (5.2)	0.012

Chronic lung disease	28 (3.9)	17 (3.7)	11 (4.1)	0.774

Psychiatric disorders	38 (5.2)	19 (4.1)	19 (7.1)	0.081

Depression	52 (7.2)	29 (6.3)	23 (8.6)	0.244

Alcohol Misuse	207 (28.5)	147 (32.0)	60 (22.5)	0.006

Drug Misuse	149 (20.5)	102 (22.2)	47 (17.6)	0.141

AIDS	3 (0.4)	2 (0.4)	1 (0.4)	0.903

Elixhauser score, mean (SD)	1.1 (5.4)	1.4 (5.6)	0.8 (4.9)	0.124

Urine Drug Screen	0 (0.0)	0 (0.0)	0 (0.0)	0.999
Amphetamines	6 (0.8)	4 (0.9)	2 (0.8)	0.863
Barbiturates	1 (0.1)	1 (0.2)	0 (0.0)	0.446
Benzodiazepines	174 (23.9)	103 (22.4)	71 (26.6)	0.201
Cocaine	109 (15.0)	51 (11.1)	58 (21.7)	<.001
Cannabinoid	152 (20.9)	93 (20.2)	59 (22.1)	0.548
Opioids				

Intervention Treatment, n (%)	237 (32.6)	157 (34.1)	80 (30.0)	0.248
Buprenorphine	105 (14.4)	77 (16.7)	28 (10.5)	0.021
Naltrexone	100 (13.8)	67 (14.6)	33 (12.4)	0.405
Methadone	342 (47.0)	200 (43.5)	142 (53.2)	0.011
N/A				

Hours to Consult Order, median (IQR)	9.2 (5.4–21.1)	8.4 (4.9–19.7)	11.7 (6.7–24.9)	<.001

Length of Stay Days, median (IQR)	4.4 (2.9–8.1)	4.2 (2.8–8.0)	4.6 (2.9–8.2)	0.785

30-Day Readmission to Same Hospital, n (%)	84 (11.6)	63 (13.7)	21 (7.9)	0.018

Any 30-Day Post-Discharge Hospital/ER Encounter, n (%)	246 (33.8)	171 (37.2)	75 (28.1)	0.013

Disposition, n (%)	610 (83.9)	391 (85.0)	219 (82.0)	0.727
Home	3 (0.4)	2 (0.4)	1 (0.4)	
Death	54 (7.4)	33 (7.2)	21 (7.9)	

LT RC / ST PA	56 (7.7)	31 (6.7)	25 (9.4)	

AMA	4 (0.6)	3 (0.7)	1 (0.4)	

Other				

Comparisons across all variables were significant with a p-value < 0.01; AMA = against medical advice; AIDS = acquired immunodeficiency syndrome; LT RC/ST PA = Long term residential care or short term post acute care; CHF = congestive heart failure; NH = non-Hispanic; Mixed = Asian, Native American or Alaskan Native, Native Hawaiian or Other Pacific Islander, Other, or Refuse/Unknown
